# A comparative ethno-botanical study of Cholistan (an arid area) and Pothwar (a semi-arid area) of Pakistan for traditional medicines

**DOI:** 10.1186/s13002-015-0018-2

**Published:** 2015-04-30

**Authors:** Sadia Malik, Saeed Ahmad, Alia Sadiq, Khurshid Alam, Hafiz Muhammad Wariss, Imtiaz Ahmad, Muhammad Qasim Hayat, Shazia Anjum, Muhammad Mukhtar

**Affiliations:** Medicinal Plant Research Laboratory, Department of Plant Biotechnology, Atta-ur-Rahman School of Applied Biosciences (ASAB), National University of Sciences and Technology (NUST), Islamabad, Pakistan; University College of Agriculture, University of Sargodha, Sargodha, Pakistan; Cholistan Institute of Desert Studies (CIDS), The Islamia University of Bahawalpur, Bahawalpur, Pakistan; Department of Biotechnology, American University of Ras Al Khaimah, PO Box 10021, Ras Al Khaimah, United Arab Emirates

**Keywords:** Medicinal plants, Traditional healthcare knowledge, Pothwar, Cholistan

## Abstract

**Background:**

The present study is intended to compare and document the therapeutic flora, their remedial use, and the traditional knowledge used frequently by the residents of the Cholistan desert and Pothwar (Potohar) Plateau of Punjab, Pakistan. The old endemic remedies of these areas are diminishing due to lack of qualitative and quantitative research.

**Methods:**

The data was generated by unstructured-interviews, informal meetings, open-ended conversations and group discussions with local people and traditional health healers of the study area. Reported literature was also utilized.

**Results:**

The study recorded a list of various medicinal plants used as traditional medicines by local people. Total 86 numbers of plant species belonging to 38 families and 67 plant species belonging to 29 families have been reported in the Pothwar and Cholistan respectively. Only 10.5% of similar plant species were present in the studied areas.

**Conclusion:**

The investigation revealed that the local people of study areas inherit a rich traditional knowledge but there is great danger of losing this wealth of knowledge in the near future. Documentation of the knowledge exclusively from desert area of Cholistan, Pakistan is unique information in its nature. The study presents the undocumented knowledge worth recognition that will not only help in conservation of medicinal plant species but will highlight the pharmacological capacity for improved human healthcare regarding many common ailments.

## Introduction

Pakistan features a diverse array of elevated peaks, snow mountains, irrigated immense plains, coasts, freezing and burning deserts. The country has been divided into four phyto-geographic regions namely the Irano-Turanian, Sino-Japanese, Saharo-Sindian and Indian element reflecting the unmatched wealth of its flora. A significant part of the country is of arid nature covering 40.9 million hectare (ha) of land together with 11 million ha of desert area. Medicinal plants play a significant role in lives of its inhabitants as they are considered as a primary source of treatment against many diseases. They also serve as a key income resource for poor field workers and people associated with herbal products manufacturers. Majority species of medicinal plants (about 70%) are uni-regional and rests of the species are bi-regional or pluri-regional [1]. The traditional healthcare knowledge has been passed on generally verbally over generations rather than as a written document due to which the knowledge is gradually diminishing. Additionally, increase in urbanization, growing population, habitat loss, improper documentation, over exploitation of some plant species, lack of implementation of laws and insufficient awareness are the factors contributing to the loss of this heritage [2].

Many workers have investigated the economic, ethno-botanic and medicinal importance of plants but ample research is still required [2-4]. However, during the past decade research work has been carried out in a range of institutions to establish the antimicrobial, anticancer, antioxidant, anti-inflammatory effects of medicinal plants [5-9]. The floristic and ethno-botanic inventories also have been made [4,10,11]. The patients mostly use allopathic, alternative and traditional medicines side by side without prescription of registered practitioners. Progress in development of an infrastructure and human resource to utilize medicinal plant wealth in a proper way is limited [4]. The ethno-botanic practices are common and have direct socioeconomic impacts [12]. All these facets are endured during this study. Study of the less explored desert region of the province also seems vital. In current study we have adopted partially the data from our previous studies [3,11].

Data regarding ethnobotanical or ethnopharmacologically characteristics of the plants of Cholistan desert and Pothwar is almost non-existent except very few reports. The main objective of present study is to explore the relationship between local culture of folk people and plants in the pursuit of drug development and medical breakthroughs. The herbal treatments in respective regions are favored over the allopathic ones for their low cost and less side effects. The most important objective of this study is the preservation of local plant knowledge. Loss of the indigenous knowledge is a threat to the poor rural economies based on traditional livestock farming as that in the deserts like Cholistan or semi-arid area like Pothwar. It was, therefore, deemed imperative to document the ethnobotany knowledge possessed by the people of respective areas. In addition to this, present study will be a yardstick to probe standardization and systematic exploration of traditional herbs.

### Study area

**Figure 1:**
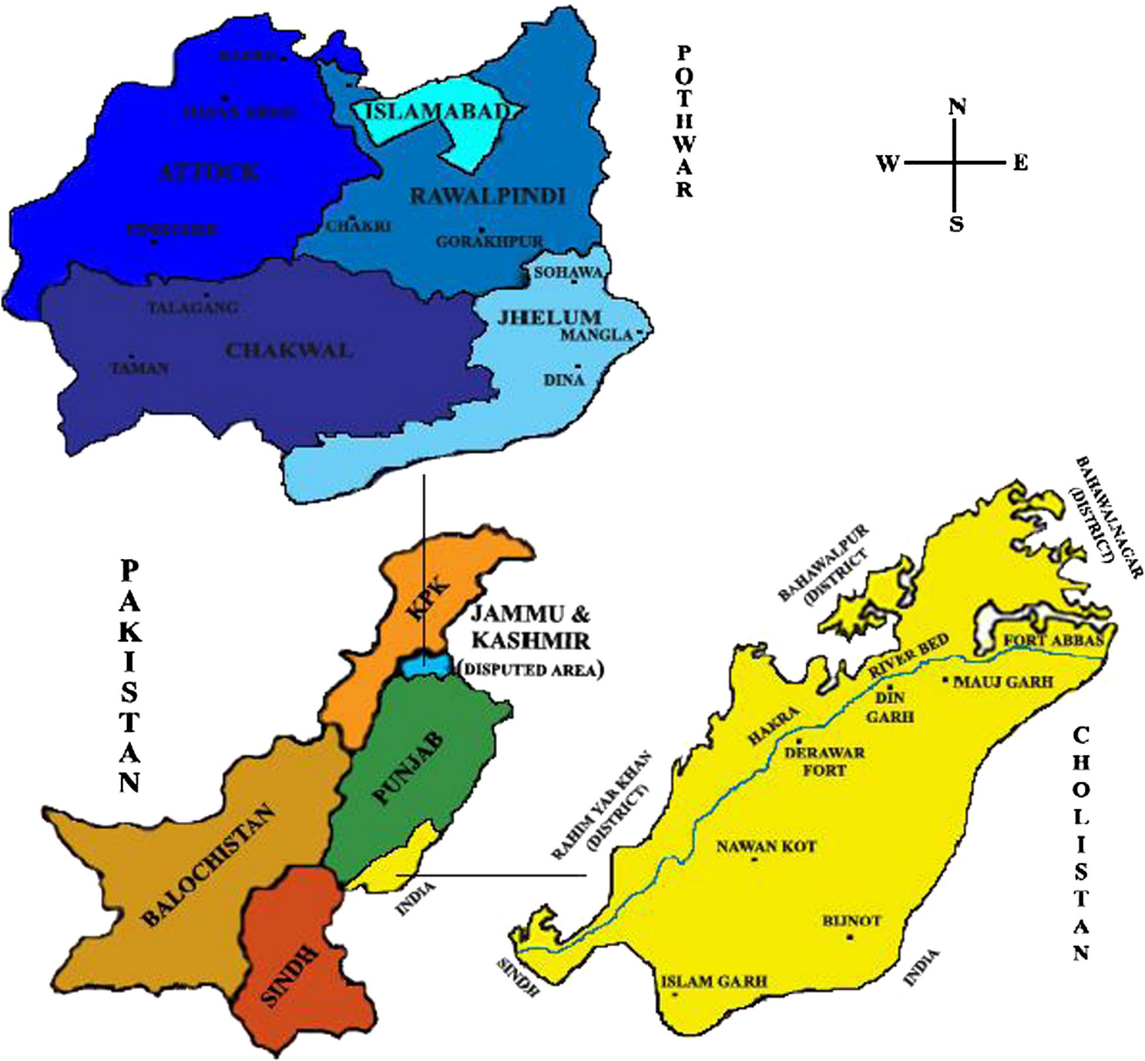
Location map of Pothwar and Cholistan areas in Pakistan.

The total geographical area of Punjab, a province of Pakistan is 20.63 million hectares with a composition of 13.37 and 7.26 million hectares as irrigated and rain fed respectively. The two areas chosen for the present study are the Pothwar (Potohar) Plateau (Semi-arid) and the Cholistan desert (arid) (Figure 1).

The Cholistan is a desert covering an area of 26,000 Km^2^ located between 27°42′ and 29°45′ N latitude and 69°52′ and 75°24′ E longitude (Figure 1) at a height of 112 m above sea level [13-16]. Its old civilization has vanished mainly due to a variety of hostile invading problems caused by the Egyptian, Harappan and Mesopotamian civilizations [17]. The prominent climatic features of the Cholistan desert are sub-tropical, arid, burning hot, monsoon rainfall with intermittent long droughts and strong summer winds having relatively low humidity and high rate of evaporation [18]. The mean annual rainfall varies between 100 mm in the West and 250 mm in the East, with heavy showers during July to September in monsoon and January to March during winter. The mean summer temperature is 34-38°C, while that of winter is 15-20°C. The June being the hottest experiences 45°C normally and sometimes rises as high as 51°C [19].

The desert is separated into two eco-regions by old Hakra River. The northern division covers about 7,770 km^2^ and is known as Lesser Cholistan. It is adjacent to canal irrigated region and progresses with a series of saline alluvial clayey flat land alternating with low sand dunes. The purely aeolian sandy desert, called the Greater Cholistan covers 8,130 Km^2^ in the southern region consisting of various forms of sand ridges and inter-ridges valleys. It extends about 480 km in length and 32 to 192 km in width. Cholistan Desert presents a multifarious prototype of alluvial and aeolian depositions [20-22].

The desert of Cholistan is one of the key ecological arid zones with extreme seasonal variation and consists of a wide variety of edaphic conditions as described above. Human population of this desert comprises of more than 110,000 pastoral nomads (originally Buddhist and Sikh, but now comprising 95% Muslims and 5% Hindu communities). The economy of the Cholistan desert is predominantly pastoral and people have been practicing a nomadic lifestyle for centuries. Saraiki is the local language of the area.

The second study area of semi-arid nature (Pothwar/Potohar) stretches from latitude 32°10-34°9 N and longitude 71°10-73°55 E comprising of Attock (Attock, Fateh Jang, Hasan abdal, Jand, Pindi Gheb), Rawalpindi (Gujar Khan, Kahuta, Kotli sattian, Murree, Rawalpindi, Taxila), Islamabad, Jehlum (Jhelum, Pind Dadan Khan, Sohawa) and Chakwal (Chakwal, Choa Saidan Shah, Talagang) districts of the Punjab [23] and covers 23,160 sq. Km area. This land tract is bordered on the north by the Kala Chitta Range and the Margalla Hills, and on the south by the Salt Range. On the eastern side lies the River Jhelum and Indus on its west. This region varies in height from 305 to 610meters above sea level and lies in the north of Salt Range. Soanian Culture prevailed in this area evidenced by the data obtained from fossils, coins, tools, and remnants of primeval archaeological sites. Historically, notable areas of this region include two UNESCO World Heritage sites, The Rohtas Fort and Taxila [24,25]. Others include namely Rawat Fort, Pharwala Fort and Katasraj temple. The typical weather of Pothwar ranges from semi-arid to sub-humid subtropical continental [26]. The Plateau is drained by Hao and Swan rivers. Its topography is tremendously varied, consisting of ridges, troughs and basins. The large part of the Plateau has been eroded and dissected by streams. The Salt Range starts from nearby Jehlum district in the Jogi Tilla and Bakrala Ridges. It crosses the Indus near Kalabagh and extends southward into the districts of Bannu and Dera-Ismaeel Khan. The average height of the range is about 671meters and the Skaser peak is 1,525meters high. Pothwar Plateau is actually an undulating, multi-colored, multi-cultural, pictorial and geologically poorly distinct area. In this densely populated land, agriculture is dependent primarily on annual rainfall (averages 380 to 510 mm). Precipitation is recorded maximum in the northwest and minimum in the southwest arid zones [27]. Various soils are found in the Pothwar Plateau typically, alluvial, loess, mixed material and colluvial types resulting from sandstone and shale [26]. Inceptisols, entisols and aridisols and with traces of alfisols soils are recorded [27]. According to 1998 district census report, 7,4,64,763 people were residing in the area and there is still tremendous increase in population. Majority of the people are Muslims while minorities include Christians, Hindus and Sikhs. The urbanity level was about 40%. However a great deal of inhabitants are still agrarian, many people are moving into industry and mining. Agricultural practices are dependent on rain fall [28]. Crop rotation and fallow are a common practice [29]. For instance wheat-ground nut cropping is a regular observation. Medicinal plants are mostly found as weeds which may compete with crops for natural resources [30] and at waste places.

The people in Punjab have their own distinct rural culture possessing their own principles, laws, traditions, ethnic groups Muslims (70%), Hindus (10%), Sikhs (15%), and Christians (5%), heritage, languages and much more. The rural to urban migration trend is also prevailing with many effects on traditions. The people in the rural areas are still highly dependent on natural products and natural remedies. They are closely related to plants and plant products play significant roles in their lives. Figure 1 shows the location map of Cholistan and Pothwar.

## Methods

The study was undertaken during different seasons of the years (2010, 2011). Total of 136 local people including traditional healers were identified using the Participatory Rapid Appraisal Approach (PRA) [31]. PRA stipulated a valuable insight into the multiple dimensions and experiences of local people with traditional plant medicines. The informal meetings, open-ended conversations allowed us to develop problems that were important to the community but unknown to the investigators [32]. With the help of the local community the most renowned traditional healers in the study area were consulted. The traditional healers were expert practitioners who medicated the local population using ethno medicinal plants and their products. The informers were 20–80 years old including both men and women (Table 1). Informal and verbal consent was taken from each individual traditional healer who took part in the study. To confirm the use of each plant used for treating the same disease, at least three traditional healers were consulted.

**Table 1:** **Age and sex characteristics of traditional healers interviewed in the present study**

**Gender**	**Age (years)**			**Total**
	**20-40**	**40-60**	**60-80**	
Male	53	38	17	108
Female	7	16	5	28

The whole study area of Cholistan was divided into 11 transect lines covering almost all macro and micro habitats. The transect lines which were followed during this study were as follows: Fort Abbas to Dodhlan Plantation Border Line Area, Dak wala to Border Line Area, Fort Morot to Rana Bhana, Kalay Pahar to Border Line Area, Yazman to Wanjuhar, Yazman to Bijnot, Fort Derawar to Bijnot, Fort Derawar to Garare Wala, Fort Khair Garh to Kakki Wala, Kot Murid to Ghunyan Wala and Rahim Yar Khan to Islam Garg while in Pothwar region, villages in Chakwal, Talagang, Jhelum, Rawalpindi, Gujar Khan, Murree, Kahuta, Pindi Gheb, Attock, Kala Chitta hills were randomly selected.

Visits were made to the places where traditional healers usually accumulate plants for their therapeutic purposes. The plants mentioned in this manuscript as traditional medicines were identified in the field by the traditional healers during the dialogues between them. Voucher specimens were assigned when plants encountered for the first time. They were also assigned during flowering or fruiting. The plant specimens were treated as per standard taxonomic procedures [33]. The collected specimens were dried in newspapers/blotting papers. The dried specimens were sprayed with a saturated solution of Mercuric Chloride in rectified spirit. The specimens were mounted very carefully on herbarium sheets of standard size (41.25 × 28.75 cm) by using German glue. All the specimens were fully labeled on the right lower corner. The field data was then given herbarium labels. The herbarium specimens of this work have been deposited in the herbarium of Cholistan Institute of Desert Studies, The Islamia University of Bahawalpur. For small herbaceous plants, the entire plants were collected. To get insight of folklore remedies and their uses and to get first hand knowledge, meetings were held with elderly inhabitants, local Hakeems (Herbal practitioners) and domestic women of Cholistan desert and Pothwar area. The interviews and deliberations were executed in Siraki, Pothwari and Punjabi, the local languages of Cholistan and Pothwar correspondingly since the authors are native speakers of the languages. Data on specific plant parts (leaves, twigs, fruits, pods stem bark, roots etc.), and the ethno-pharmacological values were collected. For the record of medicinal uses, information on the plant parts used, their collection, processing and preparation of drugs, properties, mode of administration dosage and the diseases cured were also recorded. Collected information was also verified in different localities from local inhabitants either by showing the plant specimen or telling local names to the respondents. The plants were scientifically identified and documented as well. Scientific literature already cited was also reviewed to cross check the collected information about medicinal and ethno-botanical uses.

The descriptive data gathered for this study was recorded and saved with respect to date. Contradictory and exclusive statements were noticed and were been given close attention. The repeated data was recorded in this report by data reduction technique, the systematic content analysis. In its broad sense, different reviews have highlighted a range of aspects of content analysis, from its capacity to generate quantitative descriptions by analyzing word counts [34] to its ability to help researchers infer conclusions from a text by breaking it into distinct entities of useful data that can then be implicitly rationalized that is compressing many words of text into fewer manageable groups [35].

## Results and discussion

### Medicinal plant diversity

This study records 67 plant species as useful in traditionally curing 123 human diseases in Cholistan Desert (Table 2). These medicinal plants were distributed among 29 families and 55 genera. The largest proportion of medicinal plants collected belonged to the families Zygophyllaceae (5), Capparidaceae (5), Poaceae (6), Solanaceae (3), Asclepiadaceae (4), Cucurbitaceae (3), Chenopodiaceae (4), Papilionaceae (3), Molluginaceae (3), Euphorbiaceae (3), Asteraceae (3) and Mimosaceae (3). Poaceae, Zygophyllaceae and Capparidaceae constitute the maximum diversity of species used in herbal medication.

**Table 2:** **Medicinal Flora of Cholistan Desert (Southern Punjab)**

**Sr. No.**	**Plant Name [voucher specimen #]**	**Vernacular name**	**Family**	**Plant part used**	**Disease cured**	**References**
1.	*Abutilon muticum* (Del.ex DC.) Sweet [2510/CIDS/IUB]	Kanghi-buti	Malvaceae	Leaves and roots	Renal stones and heartburn.	Traditional Health Healers [THH]; [60]
					Infectious diseases.	
2.	*Acacia jacquemontii* Benth. [2512/CIDS/IUB]	Banwli	Mimosaceae	Bark and leaves	Chickenpox, pain, fever and small pox	[THH]
3.	*Acacia nilotica* (Linn.) Delile [2513/CIDS/IUB]	Babul or Kikar	Mimosaceae	Leaves, bark, flowers, fruit and gum	Men sex problems, diarrhea, sexual debility, hemorrhages and high blood sugar.	[THH]; [61]
					Used as tonic and cure fever.	
4.	*Aerva javanica var. javanica* [2201/CIDS/IUB]	Bui	Amaranthaceae	Root, bark and leaves	Renal stones and upper-respiratory infection.	[THH]; [62]
					Constipation and remove gastrointestinal parasites.	
5.	*Alhagi maurorum* Medic [2529/CIDS/IUB]	Jawansa	Papilionaceae	Whole plant	Blood purifier, fever, Jaundice and respiratory diseases.	[THH]; [63]
					Increased perspiration, cough, and constipation.	
6.	*Blepharis scindica* T. Anders. [2001/CIDS/IUB]	Gandi-boti	Acanthaceae	Whole plant	General weakness and pain	[THH]
7.	*Boerhavia procumbens* Banks ex Roxb [2518/CIDS/IUB]	Biskhipra	Nyctaginaceae	Whole plant	Chest infections, renal failure and painful periods in women.	[THH]; [64]; [65]; [66]
					Blood purifier. Liver diseases, sexually transmitted infections and sluggishness.	
8.	*Calligonum polygonoides* Linn [2663/CIDS/IUB]	Phog	Polygonaceae	Flowers and green twigs	Sore eyes, severe thirst.	[THH]
					Indigestion, sore throat and pain.	
9.	*Calotropis procera subsp. hamiltonii* (Wight) Ali [2005/CIDS/IUB]	Ak	Asclepiadaceae	Latex, leaves, flower buds and root bark	Painful menstruation, uterus problems, asthma, stomachache and muscular weakness. Snake bite, piles, leprosy, sexually transmitted diseases, asthma and joint pain.	[THH]; [67]; [68]
10.	*Capparis decidua* (Forsskal.) Edgew [2315/CIDS/IUB]	Karir	Capparidaceae	Leaves, fruit and stem	Piles, fevers, painful menstruation, high blood sugar, obesity, indigestion and bone fractures.	[THH]; [69]
					Remove intestinal worms, used as sexual stimulant, relieves flatulence, increased perspiration and constipation.	
11.	*Capparis spinosa* Linn. [2316/CIDS/IUB]	Kubber	Capparidaceae	Leaves flowers	Joint, muscle pain, asthma, indigestion and liver disorders	[THH]
12.	*Cassia italica subsp. Italica* [2314/CIDS/IUB]	Ghoray-wall or Sana	Caesalpinaceae	Whole plant	Arthritis, pain, fevers and indigestion	[THH]
13.	*Cenchrus biflorus* Roxb. [2531/CIDS/IUB]	Mohabat Boti	Poaceae	Whole plant	Kills intestinal worms	[THH]
14.	*Cenchrus ciliaris* L. [2530/CIDS/IUB]	Daman	Poaceae	Whole plant	Intestinal worms, increase milk production in cattle and wound healing.	[THH]; [70]
					Relieves pain and used as emollient.	
15.	*Cenchrus setigerus* Vahl. [2532/CIDS/IUB	Chuti Daman	Poaceae	Whole plant	Allergies, fever, common cold and intestinal worms.	[THH]
16.	*Chrozophora sabulosa* Kar. & Kir. [2509/CIDS/IUB]	Nilakari	Euphorbiaceae	Whole plant	Leprosy, contagious infections with symptoms like cough, wheezing, cold, fever, chills, and a sore throat	[THH]
17.	*Citrullus colocynthis* (Linn.) Schrad [2350/CIDS/IUB]	Tummaor Korr- tumma	Cucurbitaceae	Roots, fruits and seeds	High blood sugar, painful menstruation, numbness or tingling in the legs and digestive disorders	[THH];[67]
18.	*Cleome brachycarpa* Vahl. ex. DC. [2317/CIDS/IUB]	Noli or	Capparidaceae	Whole plant	Intestinal worms, pain, high blood sugar, and liver disorders.	[THH]
		Kastoori				
19.	*Cleome scaposa* DC [2318/CIDS/IUB]	Khastoori boti	Capparidaceae	Whole plant	Itchiness, high blood sugar, used as diuretic and in liver disorders.	[THH]; [68]; [69]; [70]
					Reduces fever and pain.	
20.	*Convolvulus prostratus* Forssk [2348/CIDS/IUB]	Hiran-booti	Convolvulaceae	Leaves and soft twigs	Excessive thirst, heartburning, as diuretic, anti-high blood sugar and fever.	[THH]; [61]
					Treats constipation.	
21.	*Corchorus depressus* (Linn.) Stocks [2672/CIDS/IUB]	Bhaon- phali	Tiliaceae	Whole plant	Male sexual disorders and as sexual stimulant.	[THH]
22.	*Cressa cretica* Linn. [2347/CIDS/IUB]	Ooini	Convolvulaceae	Whole plant	Chest tightness and coughing, skin diseases and fever.	[THH]; [71],
					Loss of appetite, as tonic and sexual stimulant.	[72]
					Sores in mouth and ease cough.	
23.	*Crotalaria burhia* Buch.-Ham. ex Benth [2527/CIDS/IUB]	Chag	Papilionaceae	Whole plant	Reduces fever, joint pain, skin diseases, general weakness and stomachache.	[THH]; [73]
					Relieves pain in the body.	
24.	*Cucumis melo var. agrestis* Naudin [2351/CIDS/IUB]	Chibbarr	Cucurbitaceae	Fruit	Inappropriate eating habits and constipation.	[THH]
25.	*Cuscuta reflexa* Roxb. [2352/CIDS/IUB]	Akashbail	Cuscutaceae	Whole plant	Lice and dandruff, unhealthy skin and itching.	[THH]
26.	*Cymbopogon jwarancusa* (Jones) Schult.[2533/CIDS/IUB]	Katrin or Khavi	Poaceae	Whole plant	Diarrhea and vomiting, thirst, fever, joint and muscle pain. As diuretic.	[THH]
27.	*Cyperus rotundus* Linn. [2359/CIDS/IUB]	Moothaor	Cyperaceae	Roots and Deela	Indigestion, burning, excessive urination, high blood sugar, jaundice and fever. Loss of appetite.	[THH]; [74]
28.	*Dipterygium glaucum* Decne. [2319/CIDS/IUB]	Phel	Capparidaceae	Whole plant	Skin redness and irritation, wounds, unhealthy patchy skin, chronic fever.	[THH]; [75]
29.	*Echinops echinatus* Roxb. [2402/CIDS/IUB]	Unt-katara	Asteraceae	Whole plant	Jaundice, inappropriate eating habits, indigestion, Chronic liver diseases and sexual weakness.	[THH]
				and roots		
30.	*Euphorbia granulata* Forssk. [2506/CIDS/IUB]	Dudheli	Euphorbiaceae	Whole plant	Skin diseases, pain, and high blood sugar. Intestinal worms and constipation.	[THH]; [76]
31.	*Euphorbia prostrata* Ait. [2503/CIDS/IUB]	Hazar-dani	Euphorbiaceae	Whole plant	Piles, sexual weakness, skin redness and irritation and pain.	[THH]; [76]
32.	*Fagonia cretica* Linn [2675/CIDS/IUB]	Dhmasa	Zygophyllaceae	Whole plant	Liver diseases, lack of blood, fever, pain and as blood purifier and increase stamina.	(THH)
33.	*Farsetia hamiltonii* Royle [2313/CIDS/IUB]	Lathi or	Brassicaceae	Whole plant	As tonic and against stomachache and joint, muscle pain and diabetes	[THH]; [77]
		Farid- booti				
34.	*Gisekia pharnaceoides* Linn.[2101/CIDS/IUB]	Buloka-sag	Aizoaceae	Whole plant	Jaundice, inappropriate eating habits, fever and pain.	[THH]; [78]
					Constipation, remove intestinal worms and skin infections.	
35.	*Glinus lotoides* Linn [2514/CIDS/IUB]	Gandi-booti	Molluginaceae	Whole plant	Diarrhea, poor functioning of liver, externally to cure boils and wounds.	[THH]
36.	*Grewia villosa* Willd. [2673/CIDS/IUB]	Jalidar	Tiliaceae	Whole plant	Urinary tract infections, liver diseases, eye-ache, sexually transmitting diseases.	[THH]
37.	*Haloxylon recurvum* Bunge. ex. Boiss. [2335/CIDS/IUB]	Khar or	Chenopodiaceae	Whole plant	Gastric problems and kidney Stones.	[THH]
		Sajji				
38.	*Haloxylon salicornicum* (Moq.) Bunge [2336/CIDS/IUB]	Lana	Chenopodiaceae	Whole plant	Bleeding gums, Indigestion and insect stings. Prevent damage to the liver.	[THH];
						[79]
39.	*Heliotropium crispum* Desf. [2321/CIDS/IUB]	Kali-lani	Boraginaceae	Whole plant	Weakness, indigestion and laziness.	[THH]; [62]
40.	*Heliotropium strigosum subsp. Strigosum* [2311/CIDS/IUB]	Gorakh-Pan	Boraginaceae	Whole plant	Sore eyes, open wounds, sore throat, sore nipples of breasts, jaundice, used as blood purifier and to cure severe cough.	[THH]
41.	*Indigofera argentea* Burm. f. [2528/CIDS/IUB]	Neel	Papilionaceae	Whole plant	Intestinal parasites and patchy skin.	[THH]
42.	*Launaea nudicaulis* Less [2403/CIDS/IUB]	Dudhkal	Asteraceae	Whole plant	Chronic constipation and Indigestion.	[THH]
43.	*Leptadenia pyrotechnica* (Forsskal.) Decne [2002/CIDS/IUB]	Khip	Asclepiadaceae	Leaves and shoots	Abdominal cramps, constipation, painful menstruation, obesity and high blood sugar.	[THH]
44.	*Mollugo cerviana* (L.) Seringe [2515/CIDS/IUB]	Padi or	Molluginaceae	Roots	Fever, burning urination and sexually transmitted diseases.	[THH]; [80];
		Sarr			Skin itching and as blood purifier.	[64]
45.	*Mollugo nudicaulis* Lamk. [2516/CIDS/IUB]	Gandi-buti	Molluginaceae	Whole plant	Chest infection, whooping cough; leaves are applied as poultice on wounds and boils.	[THH]
46.	*Mukia maderaspatana* (Linn.) M.J. Roem [2349/CIDS/IUB]	Gawala-kakri	Cucurbitaceae	Shoots, roots and seeds	Jaundice, fever, muscular weakness and lower back pain.	[THH]
47.	*Neurada procumbens* Linn [2517/CIDS/IUB]	Chhapri	Neuradaceae	Whole plant	As sexual tonic and cure general weakness.	[THH]; [62]
					As nerve tonic.	
48.	*Oligochaeta ramosa* (Roxb.) Magenitz [2401/CIDS/IUB]	Birham dandi	Asteraceae	Whole plant	Irritation, liver diseases, joint pain and as brain tonic	[THH]
49.	*Oxystelma esculentum* (Linn. f.) R. Brown [2003/CIDS/IUB]	Dudhani	Asclepiadaceae	Whole plant	Painful urination, sexually transmitted diseases, acne and pain.	[THH]
50.	*Panicum antidotale* Retz [2534/CIDS/IUB]	Murrot or Bansi ghaa	Poaceae	Whole plant	Severe sore throat, small pox and respiratory tract infection	[THH]
51.	*Peganum harmala* Linn. [2678/CIDS/IUB]	Harmal	Zygophyllaceae	Leaves, roots and seeds	Emotional disturbances, painful menstruation, seizures, insanity and itchy skin. Abdominal pain and smoke has insecticidal properties.	[THH]; [67]
52.	*Pergularia daemia* (Jacq.) N. E. Brown. [2004/CIDS/IUB]	Karial	Asclepiadaceae	Whole plant	Intestinal worms, reduces fever, flatulence, chest tightness, stomachache and gynecological problems	[THH]
53.	*Polygonum plebejum* R. Br [2538/CIDS/IUB]	Chiri-	Polygonaceae	Roots and whole plant	Respiratory tract infections, indigestion, vomiting and diarrhea	[THH]
		Hatta				
54.	*Prosopis cineraria* (Linn.) Druce [2511/CIDS/IUB]	Jandi or Jand or kunda	Mimosaceae	Leaves, bark, flowers and pods	Heal wounds, for birth control, blood deficiency, protein deficiency, painful menstruation and joint, muscle pain.	[THH]
55.	*Pulicaria crispa* (Cass.) Benth. & Hook. f. [2339/CIDS/IUB]	Bui	Compositae	Whole plant	Fever, headache, severe cold, cough and jaundice.	[THH]
56.	*Salsola baryosma* (Roem. ex. Scult.) Dany. [2337/CIDS/IUB]	Lani	Chenopodiaceae	Whole plant	Remove intestinal worms, itching, indigestion, and sores.	[THH]
57.	*Salvadora oleoides* Decne. [2667/CIDS/IUB]	Pilu	Salvadoraceae	Fruit, bark and leaves	Nutritive deficiency, inappropriate eating habits, skin boils, high blood sugar, gum bleeding and stomachache.	[THH]
58.	*Solanum surattense* Burm. F. [2668/CIDS/IUB]	Kanderi	Solanaceae	Whole plant	Joint pain, Fever, used as blood purifier, in breathing problems, severe headache, leprosy, as diuretic, hair tonic and cure abdomen pain, gas trouble, chronic cough and pain.	[THH]; [36]; [67]
59.	*Sporobolus ioclados* (Nees ex Trin.) Nees [2535/CIDS/IUB]		Poaceae	Whole plant	Severe fever, headache and vomiting.	[THH]
60.	*Suaeda fruticosa* Forssk. ex J. F. Gmelin [2338/CIDS/IUB]	Kali lani	Chenopodiaceae	Whole plant	Constipation, painful menstruation, red eyes, indigestion and wound healing.	[THH];
						[81]
61.	*Tamarix aphylla* (Linn.) Karst [2671/CIDS/IUB]	Jhao and Ukan or Frash	Tamaracaceae	Leaves, bark and nuts	Liver diseases, indigestion, stomachache, leucorrhoea, sexual weakness and skin problems.	[THH]
62.	*Tribulus longipetalus subsp. Longipetalus* [2676/CIDS/IUB]	Tirkandi or	Zygophyllaceae	Fruits and seeds	Renal stones, male sexual problems, anemia and general weakness.	[THH]
		Bakharra				
63.	*Tribulus longipetalus subsp. Macropterus* [2677/CIDS/IUB]	Tirkindi or	Zygophyllaceae	Whole plant	Male sexual problems, itchy skin, chest/heart pain, piles, bleeding from nose and pain.	[THH]
		Bakharra				
64.	*Withania coagulens* (Stocks) Dunal [2669/CIDS/IUB]	Paneer	Solanaceae	Whole plant and fruit	Jaundice, inappropriate eating habits and skin problems.	[THH]
65.	*Withania somnifera* (Linn.) Dunal [2670/CIDS/IUB]	Asgandh	Solanaceae	Leaves and root bark	Boils, nerve weakness, joint pain and as sexual stimulant.	[THH]; [36]; [67]
66.	*Ziziphus nummularia* (Burm. f.) Wight & Arn [2664/CIDS/IUB]	Beri	Rhamnaceae	Fruit, bark, leaves and seeds	Skin diseases, cold, cough, stomachache, diarrhea, hair roughness, high blood sugar and help wound healing.	[THH]; [36]; [67]; [82]
67.	*Zygophyllum simplex* Linn [2674/CIDS/IUB]	Lunak	Zygophyllaceae	Whole plant	Patchy skin, wounds, acne and Bleeding	[THH]

Cholistan Desert is uniquely located in wild land with dearth of endemic flora counting only 128 species belonging to 32 families. During the present study people including local elders (Siana), herbal and homoeopathic practitioners and spiritual healers were interviewed. They play an imperative role in primary healthcare of the local inhabitants as the majority of their clients come from poor families who cannot meet the expense of the modern healthcare services. As said by traditional healers, the local people are still dependent on wild plants for prime healthcare owing to the widespread faith in its efficiency. According to the current survey, local people for curing various diseases, commonly use 67 plant species belonging to 29 families. The diseases cured vary from simple stomachache to more complicated such as male and female urino-genital disorders. It is evident from Table 2 that 14 plant species are being used for the treatment of gastrointestinal tract disorders. Moreover, it is observed that 16 plant species are consumed as antibacterial and cure for skin diseases. 10 of the plant species are particularly utilized for respiratory tract problems, whereas, for musculoskeletal and joint disorders 10 plant species are used. There are 5 species being consumed for the male sexual disorders, and 10 species for the female sexual disorders. For urinary tract infections 5 plant species have been exploited, and 10 plant species are being consumed as anti-diabetics. In addition to this, traditional healers are using 14 plant species to cure fever, 7 plant species to cure liver diseases, 9 plant species to treat jaundice and renal stones are being cured with 6 plant species. Five plants including *Heliotropium strigosum, Withania somnifera, Mukia maderaspatana, Cymbopogon jwarancusa, and Peganum harmala* are commonly used for the treatment of CNS disorders, like dementia.

Data acquired from Northern Punjab (Pothwar area) is assembled in Table 3 and the plants species are sorted alphabetically. A sum of 86 plant species belonging to 38 families have been reported, used for the cure of different diseases. The highest numbers (8) of medicinal plants are from Asteraceae. 22 are found to be used in treatment of jaundice and liver diseases. 22 plant species have been benefited as anti-diabetic. *Solanum surratense* has been used by local people for the cure of abdomen pain, gas trouble and chronic coughs and pain. It is also reported to possess antibacterial activity against drinking water bacteria [36,37]. Four plant species are used to cure sexually transmitted diseases, two plant species to treat sexual impotency in males, 3 being used for treatment of rheumatic/ joint pain, 7 for cough and asthma, 3 against piles, 2 species to cure urinary problems, 5 used in cure to skin problems and dandruff, 4 species for eye diseases, 4 for high blood pressure treatment, 20 for digestive system disorders, rest of the reported plants species are used against various other ailments like fever, ear pain, touch ache, few as antidote etc. In district Attock, 35 different types of human ailments that have been reported in previous studies to be used in conventional system [10].

**Table 3:** **Medicinal Flora of Pothwar Plateau (Northern Punjab)**

**Sr. No.**	**Plant Name [voucher specimen #]**	**Vernacular name**	**Family**	**Plant part used**	**Disease cure**	**References**
1	*Abutilon indicum* G. Don [0001/ASAB/NUST]	Kanghi	Malvaceae	Whole plant	Diarrhea, sexually transmitted diseases, burning with urination	Traditional Health Healers [THH]; [65]
2	*Acacia modesta* Wall. [0002/ASAB/NUST]	Phulahi	Mimosaceae	Bark of tree	gas trouble and abdominal diseases	[THH]; [67]
3	*Acacia nilotica* (L.) Delile. [0003/ASAB/NUST]	Kiker	Mimosaceae	Bark, leaves and branches	Mouth sores, gum pain and toothache, eye sores, sexual disability, diarrhea, asthma	[THH]; [67]; [68]
4	*Achyranthus aspera* L. [0004/ASAB/NUST]	Puth kanda	Amaranthaceae	Whole plant	Excessive menstruation, piles, abdominal pain, toothache, severe diarrhea.	[THH]; [65]
5	*Adhatoda vasica* Nees [0005/ASAB/NUST]	Bekkar	Acanthaceae	Leaves	High blood sugar	[THH]; [82]
6	*Adiantum capillus veneris* L. [0006/ASAB/NUST]	Sarhaj	Adiantaceae	Leaves	Jaundice and liver diseases	[THH]; [83]
7	*Albizzia lebbek* (L.) Benth [0007/ASAB/NUST]	Shrin	Mimosaceae	Leaves	Eye problems	[THH]; [67]
8	*Allium cepa* L. [0008/ASAB/NUST]	Piaz	Liliaceae	bulb	High blood pressure, high blood sugar	[THH]; [82]
9	*Allium sativum* L. [0009/ASAB/NUST]	Thoom	Liliaceae	bulb	Ear pain, hypertension, high blood sugar	[THH]; [67]; [82]
10	*Aloe vera* L. [0010/ASAB/NUST]	Knwar gandal	Liliaceae	Leaf sap, stem	Abdominal pains, constipation, skin diseases, high blood sugar	[THH]; [67]; [82]
11	*Amaranthus viridis* L. [0011/ASAB/NUST]	Chaulai	Amaranthaceae	Leaves	Menstrual disturbance, constipation	[THH]; [36]; [65]
12	*Anethum graveolense* L. [0012/ASAB/NUST]	Soye	Apiaceae	seeds	Abdominal pain	[THH]; [67]
13	*Argyrolobium roseum* (Comb) Jaub & Spach. [0013/ASAB/NUST]	Makhni	Papilionaceae	Whole plant	Jaundice and liver diseases	[THH]; [83]
		Booti				
14	*Artemisia scoparia* Walds & Kit. [0014/ASAB/NUST]	Done Jhan, pinche	Asteraceae	Whole plant	Abdominal disorder, earache	[65]
15	*Atriplex* spp. [0015/ASAB/NUST]	Gerukh pari	Chenopodiaceae	Leaves, whole plant	Fever, jaundice, slugishness, liver disease, joint pain	[36]
16	*Berberis lycium* Royle. [0016/ASAB/NUST]	Sumbul	Berberidaceae	Leaves	Jaundice and liver diseases	[THH]; [83]
17	*Boerhaavia procumbens* L. [0017/ASAB/NUST]	Itsit	Nyctaginaceae	Whole plant	Jaundice, liver diseases, sexually transmitted diseases, weakness	[THH]; [83]; [65]
18	*Bryophyllum pinnatum* Kurz. [0018/ASAB/NUST]	Zakhm-e-hayat	Crassulaceae	Leaves	Wounds healing	[THH]; [67]
19	*Cajanus cajan* (L.) Millsp. [0019/ASAB/NUST]	Arar ke dal	Papilionaceae	seeds	High blood sugar	[THH]; [82]
20	*Calotropis procera* Alton. F. [0020/ASAB/NUST]	Ak	Asclepiadaceae	Leaves, latex and flowers	Snake bite, piles, leprosy, sexually transmitted diseases, asthma, joint pain	[THH]; [67]; [68]
21	*Cannabis sativa* L. [0021/ASAB/NUST]	Bhang	Cannabinaceae	Leaves, flowers	Indigestion, sexually transmitted diseases, also used as sedative, narcotic and antidote against poison	[THH]; [36]; [65]
22	*Caralluma edulis* (L.) Benth ex Hook. f. [0022/ASAB/NUST]	Choung	Asclepidaceae	aerial parts	High blood sugar	[82]
23	*Carissa opaca* Stapf. ex. Haines [0023/ASAB/NUST]	Garanda	Apocynaceae	Leaves	Jaundice and liver diseases	[THH]; [83]
24	*Carthamus oxycantha* Bieb [0024/ASAB/NUST]	Pohli	Asteraceae	seeds	Oil is used against itching	[THH]; [65]
25	*Chenopodium album* L. [0025/ASAB/NUST]	Bathu	Chenopodiaceae	Whole plant	Jaundice, urinary problems, antidote against snake bite	[THH]; [36]; [65]
26	*Cicer arietinum* L. [0026/ASAB/NUST]	Chinnay	Papilionaceae	seeds	High blood sugar	[82]
27	*Cichorium intybus* L. [0027/ASAB/NUST]	Kasni	Asteraceae	Roots, whole plant	High blood sugar, jaundice and liver diseases	[THH]; [66]; [82]; [83]
28	*Citrullus colocynthus* (L.) Schrad [0028/ASAB/NUST]	Tumma	Cucurbitaceae	Fruit	Abdominal diseases, constipation	[THH]; [67]
29	*Convolvulus arvensis* L. [0029/ASAB/NUST]	Laili, erlai	Convolvulaceae	Aerial parts	Abdominal worms, used as deodorant, skin disorders	[THH]; [36]; [65]; [67]
30	*Cucumis sativus* L. [0030/ASAB/NUST]	Kheera	Cucurbitaceae	Fruit	Jaundice and liver diseases	[THH]; [83]
31	*Cynodon dactylon* L. [0031/ASAB/NUST]	Khabal ghas	Poaceae	Stem, leaves	Dysentery with fever	[65]
32	*Cyperus rotundas* L. [0032/ASAB/NUST]	Deela	Cyperaceae	Whole plant	Indigestion, vomiting, diarrhea and vomiting, fever	[65]
33	*Dalbergia sissoo* Roxb. [0033/ASAB/NUST]	Tali	Papilionaceae	Leaves	Dandruff	[67]
34	*Dodonaea viscosa* (L.) Jacq. [0034/ASAB/NUST]	Sanatha	Sapindaceae	Leaves	High blood sugar	[82]
35	*Eucalyptus cammaldulensis* Dehn [0035/ASAB/NUST]	Sufaida, Lachi	Myrtaceae	Leaves	Flu	[THH]; [67]
36	*Euphorbia helioscopia* L. [0036/ASAB/NUST]	Chattri dodak	Euphorbiaceae	Leaves, root, latex	Constipation, increases milk supply	[THH]; [65]
37	*Euphorbia royleana* Boiss. [0037/ASAB/NUST]	Danda thor	Euphorbiaceae	branch	Ear pain	[67]
38	*Fagonia indica* Burm. F. [0038/ASAB/NUST]	Dhamian	Zygophyllaceae	Leaves and branches	Gas trouble, skin problems, high blood sugar	[THH]; [67]; [82]
39	*Ficus bengalensis* L. [0039/ASAB/NUST]	Bohr	Moraceae	Leaves and branches latex	High blood sugar	[THH]; [82]
40	*Foeniculum vulgare* Mill. [0040/ASAB/NUST]	Soonf	Apiaceae	inflorescence	Eye-cataract, stomach disorders, indigestion	[THH]; [67]
41	*Fumaria indica* (Husskn.) H.N. Pugsley [0041/ASAB/NUST]	Shahtra papra	Fumariaceae	Whole plant	Diarrhea, also used as blood purifier	[THH]; [36]
42	*Hordeum vulgare* L. [0042/ASAB/NUST]	Jo	Poaceae	seeds	Kidney pain, high blood sugar; jaundice	[66]; [67]; [82]; [83]
43	*Ipomoea pentaphylla* (L.) Jacq. [0043/ASAB/NUST]	Kaan Kati	Convolvulaceae	Seeds	Jaundice, intestinal pain and worms	[THH]; [36]
44	*Justacia adhatoda* L. [0044/ASAB/NUST]	Bahker	Acanthaceae	roots	Jaundice and liver diseases	[THH]; [83]
45	*Kickxia ramosissima* (Wall) Janchen [0045/ASAB/NUST]	Khunger booti	Scrophulariaceae	Whole plant	High blood sugar	[82]
46	*Lactuca serriola* L. [0046/ASAB/NUST]	Kahu	Asteraceae	Whole plant	Stomach ache, cough, and asthma.	[THH]; [65]
47	*Malva parviflora* Wall. [0047/ASAB/NUST]	Sonchal	Malvaceae	Whole plant	Cough, flue and fever	[THH]; [36]
48	*Melia azedarach* L. [0048/ASAB/NUST]	Dharek, bakain, Herak	Meliaceae	Leaves, fruits	Piles, foot itching, high blood sugar, emotional disturbance, blood pressure	[THH]; [67]; [68]; [82]
49	*Momordica charantia* L. [0049/ASAB/NUST]	Karella	Cucurbitaceae	fruits	High blood sugar	[THH]; [82]
50	*Morus alba* L. [0050/ASAB/NUST]	Shehtoot, Chitta toot	Moraceae	Leaves, fruits	Cough, sore throat, jaundice and liver diseases	[THH]; [36]; [67]; [83]
51	*Morus nigra* L. [0051/ASAB/NUST]	Kalla toot	Moraceae	Fruit	Cough, sore throat, Jaundice and liver diseases	[THH]; [66]; [83]
52	*Myrsine africana* L. [0052/ASAB/NUST]	Khukan	Myrsinaceae	Leaves	Jaundice and liver diseases	[THH]; [83]
53	*Ocimum album* L. [0053/ASAB/NUST]	Chitti Tulsi	Lamiaceae	Leaves	High blood sugar	[36]
54	*Ocimum basilicum* L. [0054/ASAB/NUST]	Niazbo	Lamiaceae	Leaves	Mouth sores	[THH]; [67]
55	*Ocimum sanctum* L. [0055/ASAB/NUST]	Tulsi	Lamiaceae	Leaves	High blood sugar	[82]
56	*Oxalis corniculata* L. [0056/ASAB/NUST]	Gandora, khati booti	Oxalidaceae	Leaves, seeds	Jaundice, liver diseases, stomach disorder	[36]; [67]; [83]
57	*Parthenium hysterophorus* L. [0057/ASAB/NUST]	Chatak chandni	Asteraceae	Whole plant	Severe diarrhea.	[36]
58	*Peganum heramala* L. [0058/ASAB/NUST]	Hermal	Zygophyllaceae	Seeds and whole plant	Abdominal pain, also Smoke has insecticidal properties	[THH]; [67]
59	*Phyllanthus emblica* L. [0059/ASAB/NUST]	Aamla	Euphorbiaceae	fruits	Jaundice and liver diseases	[THH]; [83]
60	*Plantago lanceolatum* L. [0060/ASAB/NUST]	Ispaghol	Plantaginaceae	Seed husk	Gas trouble, indigestion, stomach problems	[THH]; [67]
61	*Plantago ovata* Forssk. [0061/ASAB/NUST]	Bhatti	Plantaginaceae	Fruit, Seed husk	Jaundice and liver diseases	[THH]; [66]; [83]
62	*Pongamia pinnata* (L.) Merril [0062/ASAB/NUST]	Sukh chain	Papilionaceae	Leaves, seeds, root	Skin problems and stomachache	[65]
63	*Praecitrullus fistulosus* (Stocks.) Pangalo. [0063/ASAB/NUST]	Teenda	Cucurbitaceae	leaves	Blood pressure	[67]
64	*Psidium guajava* L. [0064/ASAB/NUST]	Amrood	Myrtaceae	leaves	High blood pressure, high blood sugar, constipation	[THH]; [67]; [82]
65	*Punica granatum* L. [0065/ASAB/NUST]	Anar	Punicaceae	Fruit	Diarrhea, anemia	[THH]; [67]
66	*Raphanus sativus* L. [0066/ASAB/NUST]	Mooli	Brassicaceae	Root	Jaundice and liver diseases	[THH]; [83]
67	*Rhazya stricta* Decne. [0067/ASAB/NUST]	Vena, venra, Verian	Apocynaceae	Leaves and branches	Tooth ache, acne	[67]
68	*Ricinus communis* L. [0068/ASAB/NUST]	Arind	Euphorbiaceae	Leaves, oil	Wound healing, constipation, joints swelling and pain.	[THH]; [36]; [67]
69	*Rosa indica* L. [0069/ASAB/NUST]	Gulab	Rosaceae	petals	Eye burning, constipation, abdominal pain.	[THH]
70	*Rumex hastatus* D. Don, Prodr. [0070/ASAB/NUST]	Khatimal	Polygonaceae	Leaves	Jaundice and liver diseases	[THH]; [83]
71	*Silybum marianum* L. Gaertn [0071/ASAB/NUST]	Ount Katara, kandiali	Asteraceae	Leaves, seeds	Liver diseases, horse bite	[83]; [84]
72	*Sisymbrium irio* L. [0072/ASAB/NUST]	Khoob Kalan, Jangli sarson	Brassicaceae	Leaves, seeds	Throat, chest infection and swelling	[THH]; [36]; [65]
73	*Solanum nigrum* L. [0073/ASAB/NUST]	Kachmach	Solanaceae	Leaves and branches	Abdominal pain, stomachache, high blood sugar, burnt skin and wounds	[THH]; [37]; [67]; [82]
74	*Solanum surratense* Burm. F. [0074/ASAB/NUST]	Kandiari/Muhakri	Solanaceae	Fruits, flowers	Abdomen pain, gas trouble, chronic coughs and pain	[THH]; [36]; [67]
75	*Sonchus arvensis* L. [0075/ASAB/NUST]	Dodak	Asterceae	Whole plant	Jaundice, cough, chest stiffness, asthma	[65]
76	*Syzygium cumini* (L.) Skeets. [0076/ASAB/NUST]	Jaman	Myrtaceae	Seeds	High blood sugar	[THH]; [67]
77	*Tagetes petala* L. [0077/ASAB/NUST]	Satbarga	Asteraceae	Leaves	Ear pain	[67]
78	*Tamarindus indica* L. [0078/ASAB/NUST]	Imli	Caesalpinaceae	Fruit, Roots	Jaundice and liver diseases	[THH]; [83]
79	*Taraxacum officinale* Weber [0079/ASAB/NUST]	Doddak, Hand	Asteraceae	Leaves, Rhizome	High blood sugar, jaundice	[THH]; [82]; [83]
80	*Trachyspermum copticum* L. [0080/ASAB/NUST]	Ajwain	Apiaceae	Seeds	Gas trouble, stomach upset	[THH]; [67]
81	*Tribulus terristris* L. [0081/ASAB/NUST]	Bhakra	Zygophyllaceae	Whole plant, leaves	Joint, muscle pain, urinary disorders, impotency	[THH]; [36]; [83]
82	*Trigonella foenum-graecum* L. [0082/ASAB/NUST]	Methri	Papilionaceae	seeds	High blood sugar	[THH]; [82]
83	*Tylophora hirsuta* L. [0083/ASAB/NUST]	Glow	Asclepiadaceae	branches	High blood sugar	[82]
84	*Vigna mungo* (Burm. f.) Walp. [0084/ASAB/NUST]	Mung	Papilionaceae	seeds	High blood sugar	[82]
85	*Withania somnifera* (L.) Dunal [0085/ASAB/NUST]	Aksun, asgand	Solanaceae	leaves	Cure joint, muscle pain, uterine diseases, used as sexual stimulant.	[THH]; [36]; [67]
86	*Zizyphus nummularia* (Burm. F) Wight and Arn. [0086/ASAB/NUST]	Beri	Rhamnaceae	Leaves, fruits	Hair roughness, high blood sugar, wound healing	[THH]; [36]; [67]; [82]
**Sr. No.**	**Plant Name [voucher specimen #]**	**Vernacular name**	**Family**	**Plant part used**	**Disease Cure**	**References**
1	*Abutilon indicum* G. Don [0001/ASAB/NUST]	Kanghi	Malvaceae	Whole plant	Diarrhea, sexually transmitted diseases, burning with urination	Traditional Health Healers [THH]; [41]
2	*Acacia modesta* Wall. [0002/ASAB/NUST]	Phulahi	Mimosaceae	Bark of tree	gas trouble and abdominal diseases	[THH]; [43]
3	*Acacia nilotica* (L.) Delile. [0003/ASAB/NUST]	Kiker	Mimosaceae	Bark, leaves and branches	Mouth sores, gum pain and toothache, eye sores, sexual disability, diarrhea, asthma	[THH]; [43]; [44]
4	*Achyranthus aspera* L. [0004/ASAB/NUST]	Puth kanda	Amaranthaceae	Whole plant	Excessive menstruation, piles, abdominal pain, toothache, severe diarrhea.	[THH]; [41]
5	*Adhatoda vasica* Nees [0005/ASAB/NUST]	Bekkar	Acanthaceae	Leaves	High blood sugar	[THH]; [59]
6	*Adiantum capillus veneris* L. [0006/ASAB/NUST]	Sarhaj	Adiantaceae	Leaves	Jaundice and liver diseases	[THH]; [61]
7	*Albizzia lebbek* (L.) Benth [0007/ASAB/NUST]	Shrin	Mimosaceae	Leaves	Eye problems	[THH]; [43]
8	*Allium cepa* L. [0008/ASAB/NUST]	Piaz	Liliaceae	bulb	High blood pressure, high blood sugar	[THH]; [59]
9	*Allium sativum* L. [0009/ASAB/NUST]	Thoom	Liliaceae	bulb	Ear pain, hypertension, high blood sugar	[THH]; [43]; [59]
10	*Aloe vera* L. [0010/ASAB/NUST]	Knwar gandal	Liliaceae	Leaf sap, stem	Abdominal pains, constipation, skin diseases, high blood sugar	[THH]; [43]; [59]
11	*Amaranthus viridis* L. [0011/ASAB/NUST]	Chaulai	Amaranthaceae	Leaves	Menstrual disturbance, constipation	[THH]; [41]; [57]
12	*Anethum graveolense* L. [0012/ASAB/NUST]	Soye	Apiaceae	seeds	Abdominal pain	[THH]; [43]
13	*Argyrolobium roseum* (Comb) Jaub & Spach. [0013/ASAB/NUST]	Makhni	Papilionaceae	Whole plant	Jaundice and liver diseases	[THH]; [61]
		Booti				
14	*Artemisia scoparia* Walds & Kit. [0014/ASAB/NUST]	Done Jhan, pinche	Asteraceae	Whole plant	Abdominal disorder, earache	[41]
15	*Atriplex* spp. [0015/ASAB/NUST]	Gerukh pari	Chenopodiaceae	Leaves, whole plant	Fever, jaundice, slugishness, liver disease, joint pain	[57]
16	*Berberis lycium* Royle. [0016/ASAB/NUST]	Sumbul	Berberidaceae	Leaves	Jaundice and liver diseases	[THH]; [61]
17	*Boerhaavia procumbens* L. [0017/ASAB/NUST]	Itsit	Nyctaginaceae	Whole plant	Jaundice, liver diseases, sexually transmitted diseases, weakness	[THH]; [41]; [61]
18	*Bryophyllum pinnatum* Kurz. [0018/ASAB/NUST]	Zakhm-e-hayat	Crassulaceae	Leaves	Wounds healing	[THH]; [43]
19	*Cajanus cajan* (L.) Millsp. [0019/ASAB/NUST]	Arar ke dal	Papilionaceae	seeds	High blood sugar	[THH]; [59]
20	*Calotropis procera* Alton. F. [0020/ASAB/NUST]	Ak	Asclepiadaceae	Leaves, latex and flowers	Snake bite, piles, leprosy, sexually transmitted diseases, asthma, joint pain	[THH]; [43]; [44]
21	*Cannabis sativa* L. [0021/ASAB/NUST]	Bhang	Cannabinaceae	Leaves, flowers	Indigestion, sexually transmitted diseases, also used as sedative, narcotic and antidote against poison	[THH]; [41; [57]
22	*Caralluma edulis* (L.) Benth ex Hook. f. [0022/ASAB/NUST]	Choung	Asclepidaceae	aerial parts	High blood sugar	[59]
23	*Carissa opaca* Stapf. ex. Haines [0023/ASAB/NUST]	Garanda	Apocynaceae	Leaves	Jaundice and liver diseases	[THH]; [61]
24	*Carthamus oxycantha* Bieb [0024/ASAB/NUST]	Pohli	Asteraceae	seeds	Oil is used against itching	[THH]; [41]
25	*Chenopodium album* L. [0025/ASAB/NUST]	Bathu	Chenopodiaceae	Whole plant	Jaundice, urinary problems, antidote against snake bite	[THH]; [41]; [57]
26	*Cicer arietinum* L. [0026/ASAB/NUST]	Chinnay	Papilionaceae	seeds	High blood sugar	[59]
27	*Cichorium intybus* L. [0027/ASAB/NUST]	Kasni	Asteraceae	Roots, whole plant	High blood sugar, jaundice and liver diseases	[THH]; [42]; [59]; [61]
28	*Citrullus colocynthus* (L.) Schrad [0028/ASAB/NUST]	Tumma	Cucurbitaceae	Fruit	Abdominal diseases, constipation	[THH]; [43]
29	*Convolvulus arvensis* L. [0029/ASAB/NUST]	Laili, erlai	Convolvulaceae	Aerial parts	Abdominal worms, used as deodorant, skin disorders	[THH]; [41]; [43]; [57]
30	*Cucumis sativus* L. [0030/ASAB/NUST]	Kheera	Cucurbitaceae	Fruit	Jaundice and liver diseases	[THH]; [61]
31	*Cynodon dactylon* L. [0031/ASAB/NUST]	Khabal ghas	Poaceae	Stem, leaves	Dysentery with fever	[41]
32	*Cyperus rotundas* L. [0032/ASAB/NUST]	Deela	Cyperaceae	Whole plant	Indigestion, vomiting, diarrhea and vomiting, fever	[41]
33	*Dalbergia sissoo* Roxb. [0033/ASAB/NUST]	Tali	Papilionaceae	Leaves	Dandruff	[43]
34	*Dodonaea viscosa* (L.) Jacq. [0034/ASAB/NUST]	Sanatha	Sapindaceae	Leaves	High blood sugar	[59]
35	*Eucalyptus cammaldulensis* Dehn [0035/ASAB/NUST]	Sufaida, Lachi	Myrtaceae	Leaves	Flu	[THH]; [43]
36	*Euphorbia helioscopia* L. [0036/ASAB/NUST]	Chattri dodak	Euphorbiaceae	Leaves, root, latex	Constipation, increases milk supply	[THH]; [41]
37	*Euphorbia royleana* Boiss. [0037/ASAB/NUST]	Danda thor	Euphorbiaceae	branch	Ear pain	[43]
38	*Fagonia indica* Burm. F. [0038/ASAB/NUST]	Dhamian	Zygophyllaceae	Leaves and branches	Gas trouble, skin problems, high blood sugar	[THH]; [43]; [59]
39	*Ficus bengalensis* L. [0039/ASAB/NUST]	Bohr	Moraceae	Leaves and branches latex	High blood sugar	[THH]; [59]
40	*Foeniculum vulgare* Mill. [0040/ASAB/NUST]	Soonf	Apiaceae	inflorescence	Eye-cataract, stomach disorders, indigestion	[THH]; [43]
41	*Fumaria indica* (Husskn.) H.N. Pugsley [0041/ASAB/NUST]	Shahtra papra	Fumariaceae	Whole plant	Diarrhea, also used as blood purifier	[THH]; [57]
42	*Hordeum vulgare* L. [0042/ASAB/NUST]	Jo	Poaceae	seeds	Kidney pain, high blood sugar; jaundice	[42]; [43]; [59]; [61]
43	*Ipomoea pentaphylla* (L.) Jacq. [0043/ASAB/NUST]	Kaan Kati	Convolvulaceae	Seeds	Jaundice, intestinal pain and worms	[THH]; [57]
44	*Justacia adhatoda* L. [0044/ASAB/NUST]	Bahker	Acanthaceae	roots	Jaundice and liver diseases	[THH]; [61]
45	*Kickxia ramosissima* (Wall) Janchen [0045/ASAB/NUST]	Khunger booti	Scrophulariaceae	Whole plant	High blood sugar	[59]
46	*Lactuca serriola* L. [0046/ASAB/NUST]	Kahu	Asteraceae	Whole plant	Stomach ache, cough, and asthma.	[THH]; [41]
47	*Malva parviflora* Wall. [0047/ASAB/NUST]	Sonchal	Malvaceae	Whole plant	Cough, flue and fever	[THH]; [57]
48	*Melia azedarach* L. [0048/ASAB/NUST]	Dharek, bakain, Herak	Meliaceae	Leaves, fruits	Piles, foot itching, high blood sugar, emotional disturbance, blood pressure	[THH]; [43]; [44]; [59]
49	*Momordica charantia* L. [0049/ASAB/NUST]	Karella	Cucurbitaceae	fruits	High blood sugar	[THH]; [59]
50	*Morus alba* L. [0050/ASAB/NUST]	Shehtoot, Chitta toot	Moraceae	Leaves, fruits	Cough, sore throat, jaundice and liver diseases	[THH]; [43]; [57]; [61]
51	*Morus nigra* L. [0051/ASAB/NUST]	Kalla toot	Moraceae	Fruit	Cough, sore throat, Jaundice and liver diseases	[THH]; [42]; [61]
52	*Myrsine africana* L. [0052/ASAB/NUST]	Khukan	Myrsinaceae	Leaves	Jaundice and liver diseases	[THH]; [61]
53	*Ocimum album* L. [0053/ASAB/NUST]	Chitti Tulsi	Lamiaceae	Leaves	High blood sugar	[57]
54	*Ocimum basilicum* L. [0054/ASAB/NUST]	Niazbo	Lamiaceae	Leaves	Mouth sores	[THH]; [43]
55	*Ocimum sanctum* L. [0055/ASAB/NUST]	Tulsi	Lamiaceae	Leaves	High blood sugar	[59]
56	*Oxalis corniculata* L. [0056/ASAB/NUST]	Gandora, khati booti	Oxalidaceae	Leaves, seeds	Jaundice, liver diseases, stomach disorder	[43]; [57]; [61]
57	*Parthenium hysterophorus* L. [0057/ASAB/NUST]	Chatak chandni	Asteraceae	Whole plant	Severe diarrhea.	[57]
58	*Peganum heramala* L. [0058/ASAB/NUST]	Hermal	Zygophyllaceae	Seeds and whole plant	Abdominal pain, also Smoke has insecticidal properties	[THH]; [43]
59	*Phyllanthus emblica* L. [0059/ASAB/NUST]	Aamla	Euphorbiaceae	fruits	Jaundice and liver diseases	[THH]; [61]
60	*Plantago lanceolatum* L. [0060/ASAB/NUST]	Ispaghol	Plantaginaceae	Seed husk	Gas trouble, indigestion, stomach problems	[THH]; [43]
61	*Plantago ovata* Forssk. [0061/ASAB/NUST]	Bhatti	Plantaginaceae	Fruit, Seed husk	Jaundice and liver diseases	[THH]; [42]; [61]
62	*Pongamia pinnata* (L.) Merril [0062/ASAB/NUST]	Sukh chain	Papilionaceae	Leaves, seeds, root	Skin problems and stomachache	[41]
63	*Praecitrullus fistulosus* (Stocks.) Pangalo. [0063/ASAB/NUST]	Teenda	Cucurbitaceae	leaves	Blood pressure	[43]
64	*Psidium guajava* L. [0064/ASAB/NUST]	Amrood	Myrtaceae	leaves	High blood pressure, high blood sugar, constipation	[THH]; [43]; [59]
65	*Punica granatum* L. [0065/ASAB/NUST]	Anar	Punicaceae	Fruit	Diarrhea, anemia	[THH]; [43]
66	*Raphanus sativus* L. [0066/ASAB/NUST]	Mooli	Brassicaceae	Root	Jaundice and liver diseases	[THH]; [61]
67	*Rhazya stricta* Decne. [0067/ASAB/NUST]	Vena, venra, Verian	Apocynaceae	Leaves and branches	Tooth ache, acne	[43]
68	*Ricinus communis* L. [0068/ASAB/NUST]	Arind	Euphorbiaceae	Leaves, oil	Wound healing, constipation, joints swelling and pain.	[THH]; [43]; [57]
69	*Rosa indica* L. [0069/ASAB/NUST]	Gulab	Rosaceae	petals	Eye burning, constipation, abdominal pain.	[THH]
70	*Rumex hastatus* D. Don, Prodr. [0070/ASAB/NUST]	Khatimal	Polygonaceae	Leaves	Jaundice and liver diseases	[THH]; [61]
71	*Silybum marianum* L. Gaertn [0071/ASAB/NUST]	Ount Katara, kandiali	Asteraceae	Leaves, seeds	Liver diseases, stomach diseases	[61]; [62]
72	*Sisymbrium irio* L. [0072/ASAB/NUST]	Khoob Kalan, Jangli sarson	Brassicaceae	Leaves, seeds	Throat, chest infection and swelling	[THH]; [41]; [57]
73	*Solanum nigrum* L. [0073/ASAB/NUST]	Kachmach	Solanaceae	Leaves and branches	Abdominal pain, stomachache, high blood sugar, burnt skin and wounds	[THH]; [43]; [59]; [60]
74	*Solanum surratense* Burm. F. [0074/ASAB/NUST]	Kandiari/Muhakri	Solanaceae	Fruits, flowers	Abdomen pain, gas trouble, chronic coughs and pain	[THH]; [43]; [57];
75	*Sonchus arvensis* L. [0075/ASAB/NUST]	Dodak	Asterceae	Whole plant	Jaundice, cough, chest stiffness, asthma	[41]
76	*Syzygium cumini* (L.) Skeets. [0076/ASAB/NUST]	Jaman	Myrtaceae	Seeds	High blood sugar	[THH]; [43]
77	*Tagetes petala* L. [0077/ASAB/NUST]	Satbarga	Asteraceae	Leaves	Ear pain	[43]
78	*Tamarindus indica* L. [0078/ASAB/NUST]	Imli	Caesalpinaceae	Fruit, Roots	Jaundice and liver diseases	[THH]; [61]
79	*Taraxacum officinale* Weber [0079/ASAB/NUST]	Doddak, Hand	Asteraceae	Leaves, Rhizome	High blood sugar, jaundice	[THH]; [59]; [61]
80	*Trachyspermum copticum* L. [0080/ASAB/NUST]	Ajwain	Apiaceae	Seeds	Gas trouble, stomach upset	[THH]; [43]
81	*Tribulus terristris* L. [0081/ASAB/NUST]	Bhakra	Zygophyllaceae	Whole plant, leaves	Joint, muscle pain, urinary disorders, impotency	[THH]; [57]; [61]
82	*Trigonella foenum-graecum* L. [0082/ASAB/NUST]	Methri	Papilionaceae	seeds	High blood sugar	[THH]; [59]
83	*Tylophora hirsuta* L. [0083/ASAB/NUST]	Glow	Asclepiadaceae	branches	High blood sugar	[59]
84	*Vigna mungo* (Burm. f.) Walp. [0084/ASAB/NUST]	Mung	Papilionaceae	seeds	High blood sugar	[59]
85	*Withania somnifera* (L.) Dunal [0085/ASAB/NUST]	Aksun, asgand	Solanaceae	leaves	Cure joint, muscle pain, uterine diseases, used as sexual stimulant.	[THH]; [43]; [57]
86	*Zizyphus nummularia* (Burm. F) Wight and Arn. [0086/ASAB/NUST]	Beri	Rhamnaceae	Leaves, fruits	Hair roughness, high blood sugar, wound healing	[THH]; [43]; [57]; [59]

Data acquired from Northern Punjab (Pothwar area) and Southern Punjab (Cholistan) bears only 10.5% similarity. The similar medicinal plants of both areas are comprised of 9 plant species such as *Calotropis procera* subsp. *hamiltonii* (Wight) Ali, *Citrullus colocynthis* (Linn.) Schrad, *Cyperus rotundus* Linn., *Acacia nilotica* (Linn.) Delile, *Boerhavia procumbens* Banks ex Roxb, *Ziziphus nummularia* (Burm. f.) Wight & Arn., *Solanum surattense* Burm. F., *Withania somnifera* (Linn.) Dunal and *Peganum harmala* Linn.

### Plant parts used

**Figure 2:**
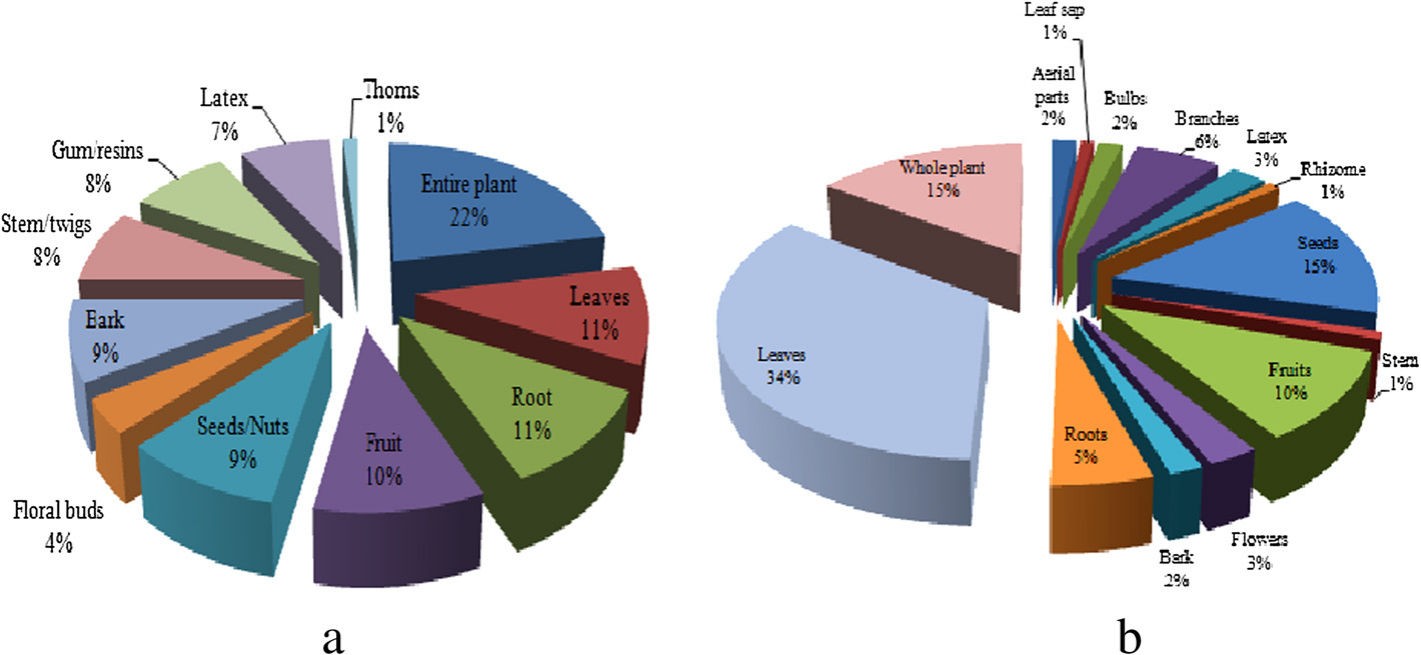
Plant parts (%) used as medicine against different diseases in **a**. Cholistan Desert and **b**. Pothwar Plateau.

Plants as whole and different parts are commonly used to cure different diseases in the study area. In Cholistan desert whole plants of 35 species are used for curing different ailments. Leaves of 17 plants are used to prepare different medicines. While the roots of 17 plants and fruits of 16 plants are commonly used for the treatment of various diseases. Seeds/nuts of 15 plants bark 14 and are being used to treat several ailments. Stems/twigs of 13 plants are commonly used, whereas gum/resin 13, latex 12, floral buds of 6 plants and thorns of 2 plants are used as medicine. Among all the plants studied in Pothwar Plateau leaves of most plants (39) are used in herbal remedies to cure different ailments while whole plants and seeds of 17 of these are mostly used medicinally by local inhabitants. Percentage of all plants parts used is depicted in Figure 2.

Twenty eight therapeutic plants are used in the form of decoction and paste (used externally). 25 plant species are used in the form powder and syrup of 12 plant species are prepared before their use as medicine. Infusion of 11 plant species, fresh juice of 8 plant species and tablets of 7 plant species are prepared and used as medicine. “Majoon” /electuary of 7 plant species, ash or salt of 5 plant species and mother tincture of 4 plants are used to cure different diseases in Cholistan Desert areas.

### Diagnosis and treatment of diseases

Traditional healers commonly diagnose illness by visual inspection, interviewing patients for symptoms and the duration of the health problem. Symptoms related to variation in pulse rate, eye color, skin color, mouth infections, body temperature and condition of sores, are the basis for prescription of remedy. Internal disorders are usually cured by recommending the herbal preparations in the form of syrup, while external wounds and dermal infections are treated by applying and rubbing herbal preparations on the affected parts.

Ethno-pharmacologically prepared drugs are traditionally administered with either medium like water, cow/buffalo milk, goat milk, sheep milk, “*lassi*” and “*arq*” (distillate). These media are commonly advised according to the condition of the patient, age and nature of the disease. The main purpose of these liquids is for good absorption of medicine, to minimize the side effects (if any) of given remedy, and sometimes these are recommended to provide the nutritional support to the patient along with medicines. During present study it has been observed that water is recommended for medicine preferably than followed by cow/buffalo milk, goat milk, sheep milk, “*lassi*” and “*arq*”.

The comprehensive information on ethno-medicinal flora of Cholistan desert and Pothwar with regard to scientific names, local names, family, part used and diseases cure have been provided in Tables 2 and 3 respectively.

The survey of both areas revealed the variation in recommended dosages of medicinal plants among the traditional healers for treating the same disease. The traditional healers believed that the doses for liquid preparations are prescribed in terms of a full, half or one fourth of a cup, depending on the age, physical condition of the patient being treated, type of illness, diagnosis and severity of the disease. There is no standardized criterion for the dosage of herbal remedies. The quantities of preparation and prescription rates generally vary with the degree and duration of the ailment. The age group of the patient, type and level of disease further decide the rate of treatment. Lack of standard procedures and quality control is seen as a common problem of conventional medicines in the developing world [38].

During the recent past ethnobotanic research has been done tremendously to explore, use and conserve natural resources especially in search of novel crude drugs. Medicinal plants are a significant source of phytochemicals that are of great importance for the health of individuals and communities [38]. Geographically quite many ethnobotanic studies have been published in recent years in Pakistan [2]. Nevertheless some of them are mentioned here. During past 5 years most of the studies are done in mountain regions [39-43]. The studies are mainly focused on listing of ethnobotanic uses of plants. Some of the plants reported have also been proven scientifically to contain valuable medicinal properties [44-48]. Several ethnobotanic surveys done from Punjab province include Soan Valley [49], Thal desert [50], Khusab [51] and Sialkot [52]. Specific studies done on Cholistan area include [53-59]. The present study also supports the usages reported by earlier studies cited in Tables 2 and 3.

## Conclusions and recommendations

The reported number of medicinal plants (67 species) in the native folklore remedies seems very significant considering the vegetation cover of Cholistan as the area has very sparse vegetation. The prolonged and reoccurring drought phenomenon, environmental degradation, grazing pressure and wood cutting for fuel purpose are the notable factors worth consideration to assess the significance of medicinal plants of Cholistan.

It can be concluded from the present study that Cholistan desert and arid areas of Pothwar (86 Species) are rich in indigenous medicinal plant wealth. Data acquired on medicinal plants of Northern Punjab (Pothwar area) and Southern Punjab (Cholistan) bears only 10.5% similarity. The total number of population in the area also justifies the high number of plants species under medicinal use. The local people of both areas possess a wealth of traditional knowledge. Plants of semiarid area of Pothwar have comparatively been explored more scientifically. The present information provides basis for the recognition of this undocumented knowledge but will also help in conservation of such an important desert species of Cholistan. It also opens the way for further investigation in new dimensions for better healthcare of human being regarding various diseases. This study has highlighted that old indigenous knowledge of traditional medicine is still in use predominantly in Cholistan desert. Many folk remedies used in traditional medicine are used as a first line of health care. The trust of the respondents on traditional medicine regarding its efficacy and cost effectiveness establishes its preference over the modern allopathic drugs. But there is variation in use of these folk remedies in both areas. The faith of the Cholistani pastorals is more in the traditional medicines. As the folklore knowledge has been passed on generally verbally from generation to generation and not documented, it is gradually fading. So it is recommended that research and developmental efforts should be focused on these plants species to scientifically identify the plant potential and substantially improve the traditional herbal therapies of the rural people.

The observations emerged from the present study should be substantiated with pharmaco-chemical studies in order to evaluate their effectiveness. The study indicated that the area of study have plenty of medicinal plants to treat a wide range of human ailments. Present study revealed that the local people prefer folk medicine due to low cost and sometimes it is a part of their social life and culture. It develops proactive link between short-term actions to long-term goals and offers analytical tools to the survival of human communities. Therefore, it is imperative to acquire and preserve this traditional system of plant utilization by proper documentation and identification of specimen. This work presents a review focusing on the main historical and current interactions between humans and the flora, the ecological implications and the role of the ethnobotany in plants conservation. The importance of ethnobiological studies for biodiversity conservation has increasingly been recognized. Sustainable harvesting of these plants is an essential component of this study to conserve natural sources. Thus there is a need to create awareness about the importance of these plants among local people and to provide them guidance and training in collection and processing to enhance the economic benefits from their indigenous flora.
